# Comparison of the Effects of Diuretics on Pedal Edema in Patients with Cancer

**DOI:** 10.1089/pmr.2022.0017

**Published:** 2022-08-18

**Authors:** Sari Nakagawa, Kouhei Tsuji, Shouhei Ishida, Nobuko Tsunoda, Yoshiaki Okamoto

**Affiliations:** ^1^ Department of Clinical Pharmacy, Faculty of Pharmaceutical Sciences, Kobe Gakuin University, Kobe, Japan.; ^2^ Department of Pharmacy, Ashiya Municipal Hospital, Ashiya, Japan.

**Keywords:** diuretics, foot edema, mineralocorticoid receptor antagonists, patients, pedal edema, physician

## Abstract

**Background:**

The effectiveness of tolvaptan (T) for treating pedal edema remains unknown.

**Objective:**

We aimed to clarify the effectiveness of diuretics, including T, on pedal edema in advanced cancer patients, and to compare patients' versus physicians' assessments of the effects.

**Methods:**

Participants comprised 88 hospitalized cancer patients treated with T, loop diuretics (L), or spironolactone (S). Patient characteristics, initial doses of diuretics, reason for discontinuation, side effects, evaluation of pedal edema, and effects of diuretics on pedal edema were investigated retrospectively using electronic medical records.

**Results:**

The rates of improvement of pedal edema according to patients (Pt) and physicians (MD) were T: Pt 83.3% (*n* = 6), MD 71.4% (*n* = 14); L: Pt 57.1% (*n* = 14), MD 50.0% (*n* = 26); S: Pt 0% (*n* = 1), MD 57.1% (*n* = 7); L+S: Pt 83.3% (*n* = 12), MD 69.0% (*n* = 29); T+L: Pt 90.9% (*n* = 22), MD 71.8% (*n* = 39); T+S: Pt 0% (*n* = 1), MD 0% (*n* = 2); T+L+S: Pt 62.5% (*n* = 8), MD 69.2% (*n* = 13). In 57.1%–90.9% and 50.0%–71.8% of episodes, patients and physicians, respectively, observed some effectiveness of diuretics on pedal edema in advanced cancer, except for in the S (Pt) and T + S (Pt, MD) groups.

**Conclusions:**

The treatment of pedal edema improves patient symptoms, enhancing quality of life. Further verification and evaluation of the effect of T on pedal edema are needed.

## Introduction

Edema is a common symptom in patients with advanced cancer. Edema is not directly related to a patient's prognosis. The reported prevalence of edema is 11% in the palliative care population.^1^ There are various primary causes, including stenosis or occlusion of the subclavian artery, superior vena cava, or iliac vein owing to a tumor or metastatic lymph node, carcinomatous lymphangiosis, or disuse syndrome resulting from motor neuropathy or carcinomatous pain.^2^ Edema may appear anywhere in the body, but it is most common in the hands and feet.

Pedal edema may occur physiologically, for example, because of long hours spent standing at work. There are also cases in whom pedal edema appears in patients with advanced cancer due to poor circulation caused by a weakening of the leg muscles; failure of the heart, kidneys, or liver; malnutrition; low protein; or decreased thyroid function.^3–5^

Edema itself is not related to the patient's prognosis; however, when accompanying advanced cancer, as the cancer worsens, symptoms of edema appear and induce suffering for both the patients and their family. Pedal edema directly results in decreased quality of life (QOL) as patients feel discomfort and may have difficulty walking or an increased risk of falling, depending on the severity. Patients with advanced cancer who experience edema are likely to have poor QOL and often suffer from pain (67%) or skin tightness (43%) in the affected limb.^6^ Therefore, improving these symptoms, even slightly, is linked to improvement in patient QOL.

Nonetheless, managing pedal edema is difficult, because it does not respond well to treatment. To date, various diuretics have been used to mitigate edema. In September 2013, tolvaptan (T) became the first therapeutic agent in the world to be approved for use against fluid accumulation in cirrhosis of the liver, for which other diuretic drugs, such as loop diuretics (L), are ineffective. T is an arginine vasopressin type 2 (V2) receptor antagonist (aquaretics)—is a new therapeutic agent. Vasopressin reduces the amount of water excreted from the kidneys, thereby retaining more water, and lowering the sodium concentration in the body.

Vasopressin also maintains fluid homeostasis by binding to V2 receptors in the renal collecting tubule. T suppresses the reabsorption of water from urine to the blood by selectively inhibiting the binding of vasopressin to V2 receptors; thus, only water is discharged from the body, without directly affecting the discharge of electrolytes such as sodium.^7–9^ T has been approved for use in fluid retention in heart failure and cirrhosis, hyponatremia in syndrome of inappropriate secretion of antidiuretic hormone, and autosomal dominant multiple cystic kidneys. Presently, its use in advanced cancer constitutes off-label drug use in Japan. This drug is approved for use conditionally: its use must be started during hospitalization and in combination with aldosterone antagonists and L—the main constituents of conventional drug treatments.

This treatment is expected to be highly effective in improving hyponatremia and ascites^10–12^ and is currently used in many facilities. However, currently, no studies have investigated the effectiveness of T on pedal edema in advanced cancer patients. Therefore, we investigated the effect of diuretics, including T, both independently and in combination, on pedal edema in advanced cancer patients. The primary aim of this study was to clarify the effectiveness of diuretics on pedal edema in advanced cancer patients by patients' and physicians' assessments of the effects. The secondary aim was to reveal whether there was a difference between patients' subjective assessments and physicians' objective assessments of the effectiveness of diuretics for edema treatment and to confirm the onset of side effects due to diuretics.

## Materials and Methods

### Study design

This was a retrospective single-facility study.

### Participants

Potential participants comprised 118 hospitalized patients who were treated between March 2015 and March 2017 for cancer with T; L, including furosemide (F) or azosemide (A); or spironolactone (S). Patients had received only one treatment (independently) or a combination of treatments: L+S, T+S, or T+S+L. The exclusion criteria were having no data on administration start date, duration of drug administration fewer than three days, and no data on area of edema. Patients were also excluded if the diuretics were given for anything other than pedal edema or if the treatment administration route differed from “oral.” We screened 118 potential participants, of whom 30 were ineligible for participation. Thus, ultimately 88 participants were included in the study.

### Measures

Patient characteristics (age, gender, primary cancer site); initial doses of diuretics; intake duration of the three diuretics; reason for discontinuation; side effects (dry mouth, increased urinary frequency); evaluation of pedal edema of each drug use episode from the commencement until the end (patients' subjective assessment, physicians' objective assessment); and effects of diuretics on pedal edema were retrospectively investigated using electronic medical records in the hospital information system. Electronic medical records were written in SOAP format (Subjective data, Objective data, Assessment, Plan). Patients' evaluations were collected from the subjective data description in the electronic medical record.

Physicians' evaluations were collected from the description of objective data or assessment in the physician's record. Patients' and physicians' evaluations were collected individually from different sources. A diuretic was considered effective when electronic medical charts indicated either patients' claims of improvement in pedal edemas or physicians' confirmation of such improvement. Diuretic ineffectiveness was also investigated as both the patients' subjective assessment and the physicians' objective assessment.

In these assessments, a diuretic was considered ineffective if either the patient claimed that the pedal edema had worsened/not improved or the physician confirmed that the pedal edema had worsened/not improved according to the electronic medical chart records. Assessment of the effect of diuretics included episodes of discontinuation and resumption in the same patient. Regarding the effect of the drug, all the descriptions in the electronic medical record from the start date of the diuretics to the 8th day were confirmed. Reasons for discontinuation and side effects (dry mouth, increased urinary frequency) were collected from the description of objective data or assessment in the physician's record.

### Statistical methods

We analyzed the patient characteristics and comparison between the drugs used, considering the overlapping effects of the drugs. A general linear model analysis was performed for age, which was continuous data. For the independent variable, we built a model wherein each drug use was inputted at the same time. Gender and primary cancer site were analyzed using a generalized linear model (link function was logit).

A generalized linear mixed model (link function was logit) with variable factors as subjects was analyzed for the onset of dry mouth, increased urinary frequency, and effects of diuretics on pedal edema. The analysis target was an episode unit. For the independent variable, we built a model wherein each drug use was inputted at the same time.

For the evaluation of pedal edema, the degree of agreement between the patients' subjective assessment and the physicians' objective assessment was evaluated by calculating kappa statistics. Values of kappa >0.80 indicated very good agreement; between 0.61 and 0.80, good agreement; 0.41 and 0.60, moderate agreement; 0.21 and 0.40, fair agreement; and 0.20, poor agreement.

## Results

### Patient characteristics

Of the 118 potential participants in the study, 30 were disqualified by the exclusion criteria and 88 participants remained. The patient characteristics are given in Table 1. Diuretics were used for 142 episodes during the survey period. Assessment of the effect of diuretics included episodes of discontinuation and resumption in the same patient.

**Table 1.** Patient Characteristics

		All	Diuretics			*p*		
			T group^a^	L group^b^	S group^c^	T group^a^	L group^b^	S group^c^
Total patients (total number of drug used, episodes^d^)		88 (142)	48 （73）	77 (118)	43 (58)			
Age	Mean ± SD	74.5 ± 14.4	74.3 ± 12.5	75.4 ± 14.3	73.3 ± 15.6	0.238	0.220	0.178
Gender, *n* (%)	Male: Female	43 (48.9): 45 (51.1)	23 (47.9): 25 (52.1)	39 (50.6): 38 (49.4)	22 (51.2): 21 (48.8)	0.963	0.385	0.719
Cancer site,^e^ *n* (%)	Liver	35 (39.8)	27 (56.3)	29 (37.7)	18 (41.9)	<0.001	0.210	0.100
	Lung	18 (20.5)	9 (18.8)	14 (18.2)	4 (9.3)	0.148	0.129	0.007
	Pancreas	13 (14.8)	10 (20.8)	10 (13.0)	7 (16.3)	0.050	0.154	0.248
	Colon	13 (14.8)	10 (20.8)	11 (14.3)	2 (4.7)	0.294	0.906	0.040
	Stomach	10 (11.4)	5 (10.4)	10 (13.0)	5 (11.6)	0.759	0.999	0.987
	Other	24 (27.3)	8 (16.7)	24 (31.2)	15 (34.9)	0.025	0.999	0.260

^a^ T group includes patients who used only T and patients who used a combination (e.g., T and L (L; T+L), T and S (S; T+S), or T+L+S.

^b^ L group includes patients who used only L and patients who used a combination (e.g., T+L, L+S, T+L+S).

^c^ S group includes patients who used only S and patients who used a combination (e.g., L+S, T+S, T+L+S).

^d^ The total number of patients in the study was 88. Diuretics were used for 142 episodes during the survey period. Assessment of the diuretic effects included episodes of discontinuation and resumption in the same patient.

^e^ Some patients had multiple primary cancer sites.

L, loop diuretics; S, spironolactone; SD, standard deviation; T, tolvaptan.

Among the patients treated with T, there were statistically significantly more patients with liver cancer and fewer with other cancers. Among those treated with S, there were statistically significantly fewer patients with colorectal and lung cancer.

### Initial doses and intake duration of diuretics

The mean (±standard deviation [SD]) of the initial doses of T group (*n* = 73) was 9.8 ± 3.9 mg; F group (*n* = 85), 26.3 ± 12.7 mg; A group (*n* = 38), 41.8 ± 14.9 mg; and S group (*n* = 58), 32.3 ± 11.5 mg. The median (min–max) of the initial doses of T group was 7.5 (3.75–15) mg; F group, 20 (5–60) mg; A group, 30 (30–60) mg; and S group, 25 (25–50) mg.

The mean (±SD) of the intake duration of T group (*n* = 65, no data [ND] = 8) was 26.4 ± 29.8 days; F group (*n* = 71, ND = 14), 27.5 ± 47.1 days; A group (*n* = 29, ND = 9), 28.7 ± 33.2 days; and S group (*n* = 49, ND = 9), 27.5 ± 28.6 days. The median (min–max) of the intake duration of T group was 13 (2–126) days; F group, 16 (2–375) days; A group, 14 (3–126) days; and S group, 16 (2–108) days. Regarding the intake duration of the diuretics, ND was used when the patient was discharged or transferred, and it was unclear whether the patient continued treatment.

### Reasons for discontinuation of diuretics

The reasons for discontinuation are given in Table 2. The group using T had more discontinuation of diuretics due to resolution of edema than the other groups.

**Table 2.** Reasons for Discontinuation

	Total number of drug uses^a^ episodes	Edema resolved^b^ ***n*** (%)	Drug ineffective^b^ ***n*** (%)	Adverse events suspected^b^ ***n*** (%)	Discharge^b^ ***n*** (%)	Death^b^ ***n*** (%)	Other^b^ ***n*** (%)	ND^c^ ***n*** (%)
T group	73	7 (9.6)	0 (0.0)	3 (4.1)	6 (8.2)	37 (50.7)	3 (4.1)	17 (23.3)
L group	114	5 (4.4)	2 (1.8)	7 (6.1)	18 (15.8)	40 (35.1)	4 (3.5)	38 (33.3)
S group	57	3 (5.3)	1 (1.8)	5 (8.8)	11 (19.3)	17 (29.8)	3 (5.3)	17 (29.8)
T only	14	3 (21.4)	0 (0.0)	0 (0.0)	1 (7.1)	5 (35.7)	0 (0.0)	5 (35.7)
L only	25	0 (0.0)	1 (4.0)	0 (0.0)	5 (20.0)	3 (12.0)	1 (4.0)	15 (60.0)
S only	8	1 (12.5)	0 (0.0)	1 (12.5)	2 (25.0)	2 (25.0)	1 (12.5)	1 (12.5)
L + S	32	1 (3.1)	1 (3.1)	4 (12.5)	8 (25.0)	6 (18.8)	1 (3.1)	12 (37.5)
T + L	42	3 (7.1)	0 (0.0)	3 (7.1)	4 (9.5)	23 (54.8)	1 (2.4)	8 (19.0)
T + S	2	0 (0.0)	0 (0.0)	0 (0.0)	0 (0.0)	1 (50.0)	0 (0.0)	1 (50.0)
T+L+S	15	1 (6.7)	0 (0.0)	0 (0.0)	1 (6.7)	8 (53.3)	2 (13.3)	3 (20.0)

^a^ Four episodes in which the use of diuretics was continued (not stopped) were excluded.

^b^ These were collected from the description of objective data or assessment provided in the physician's record.

^c^ There are no descriptions of the reasons for discontinuation in the electronic medical records.

### Total occurrence of dry mouth and increased urinary frequency

Total occurrences of dry mouth and increased urinary frequency are given in Table 3. The T-user group tended to have a statistically significantly higher frequency of dry mouth than other groups.

**Table 3.** Total Occurrence of Dry Mouth and Increase of Urinary Frequency

	Total number of drug uses	Frequency, ***n*** (%)	OR to frequency		
			OR	95% CI	***p***
Dry mouth^a^					
T	73	27 (37.0)	4.154	1.582–10.909	0.004
L	118	33 (28.0)	2.676	0.754–9.506	0.127
S	58	12 (20.7)	0.992	0.378–2.603	0.987
Increase of urinary frequency^a^					
T	73	16 (21.9)	1.001	0.393–2.549	0.998
L	118	25 (21.2)	1.343	0.393–4.591	0.636
S	58	8 (13.8)	0.509	0.189–1.372	0.180

^a^ These were collected from the description of objective data or the assessment provided in the physician's record.

CI, confidence interval; OR, odds ratio.

### Evaluation of pedal edema

**FIG. 1.**
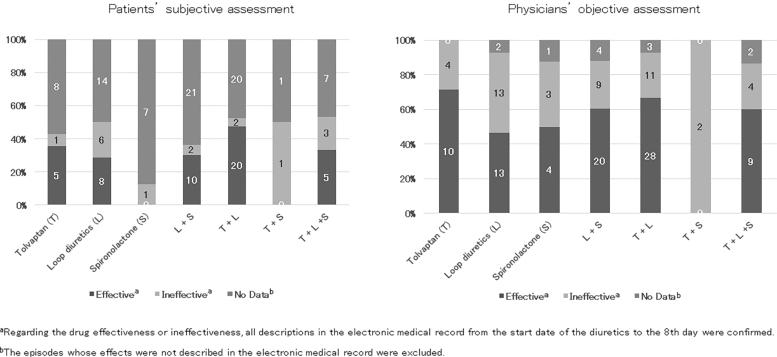
Evaluation of pedal edema.

The evaluations of pedal edema by the patients and physicians are shown in Figure 1. Physicians' assessments in 91.5% of episodes (130 of 142) were confirmed using electronic medical records; however, patients' assessments were confirmed in only 45.1% of episodes (64 of 142). Episodes whose effects were not described in the electronic medical record were excluded (patients' assessment: 78 episodes of 142 episodes, physicians' assessment: 12 episodes of 142 episodes).

The rates of improvement in pedal edema, excluding the ND data, according to patients (Pt) and physicians (MD), were T: Pt 83.3% (*n* = 6), MD 71.4% (*n* = 14); L: Pt 57.1% (*n* = 14), MD 50.0% (*n* = 26); S: Pt 0% (*n* = 1), MD 57.1% (*n* = 7); L+S: Pt 83.3% (*n* = 12), MD 69.0% (*n* = 29); T+L: Pt 90.9% (*n* = 22), MD 71.8% (*n* = 39); T+S: Pt 0% (*n* = 1), MD 0% (*n* = 2); and T+L+S: Pt 62.5% (*n* = 8), MD 69.2% (*n* = 13).

The evaluations of the degree of agreement between the physician and patient assessment are given in Table 4. The results of the analysis showed high kappa statistics (0.833), indicating a high agreement between the patients' subjective assessment and the physicians' objective assessment.

**Table 4.** Evaluation of the Degree of Agreement between the Physician and Patient

	Physicians' objective assessment	
	Ineffective^a^	Effective^a^
Patients' subjective assessment		
Ineffective^a^	15	1
Effective^a^	2	45

Kappa statistics: 0.877.

The episodes whose effects were not described in the electronic medical record were excluded (patients' assessment: 69 episodes, physicians' assessment: 7 episodes.)

^a^ Regarding the drug effectiveness or ineffectiveness, all descriptions in the electronic medical record from the start date of the diuretics to the 8th day were confirmed.

### Effects of diuretics on pedal edema

The effects of diuretics on pedal edema are given in Table 5. Although no statistically significant difference was observed, patients and physicians tended to notice an effect when using T in many episodes.

**Table 5.** Effects of Diuretics on Pedal Edema

	Total number of drug use^a^ episodes	Effective^b^ ***n*** (%)		OR to effective			
				OR	95% CI		*p*
Patients' subjective assessment							
T	37	30	(81.1)	2.628	0.675,	10.228	0.102
L	56	43	(76.8)	3.074	0.486,	19.465	0.097
S	22	15	(68.2)	0.682	0.178,	2.610	0.344
Physicians' objective assessment							
							
T	68	47	(69.1)	1.794	0.727,	4.428	0.195
L	107	70	(65.4)	1.456	0.505,	4.195	0.789
S	51	33	(64.7)	1.292	0.522,	3.197	0.919

^a^ The episodes whose effects were not described in the electronic medical record were excluded (patients' assessment: 78 episodes of 142 episodes, physicians' assessment: 12 episodes of 142 episodes).

^b^ Regarding the drug effectiveness or ineffectiveness, all descriptions in the electronic medical record from the start date of the diuretics to the 8th day were confirmed.

## Discussion

Presently, various diuretics are used to mitigate edema, but there is no research comparing their effects on pedal edema, and there is little evidence of diuretics improving pedal edema. Regarding the primary outcome of this study—the effectiveness of diuretics on pedal edema—effectiveness was observed by patients and physicians in 57.1%–90.9% and 50.0%–71.8% of episodes, respectively. Regarding the secondary outcome—differences in assessments between physicians and patients—the statistical analysis indicated that the patients' and physicians' evaluations largely matched.

The objective of edema treatment is to improve patient symptoms, resulting in improved QOL, and mitigate related risks. Therefore, it is imperative that symptoms improve according to the patients' own subjective assessment. Thus, it is necessary to introduce an assessment of patient QOL and evaluate the effectiveness of diuretics for pedal edema improvement. In this study, some patients developed dryness of the mouth mucosa after starting diuretics. Treatment with diuretics may cause such side effects; in such cases, it is better to consider not only the use of diuretics, but also nonpharmacological therapies, such as compression therapy.^13^

Although worsening of kidney function is the most serious problem associated with conventional diuretics, T has a minimal effect on kidney function and can be expected to improve ascites, even with hypoalbuminemia, which is difficult to treat with conventional L diuretics.^8,10^ Included in the Japanese treatment guidelines for cirrhosis of the liver, revised in 2020,^14^ T is also markedly effective as part of the treatment strategy for cirrhotic ascites.^11^

Although we found no statistically significant differences in the effectiveness of the various drugs for treating pedal edema, the ratio of patients with improvement of pedal edema was relatively high—the rates of effectiveness determined by patients and physicians were 62.5%–90.9% and 69.2%–71.8%, respectively—for T monotherapy and combinations of T with other diuretics. Regarding reasons for discontinuation, the T group had more discontinuation of diuretics due to resolved edema than the other groups. Hereafter, it is necessary to collect additional data to further evaluate and clarify the effects of T on pedal edema.

### Limitations

This study is limited in that it was a retrospective investigation at a single facility. Furthermore, only a small number of patients were treated with T, either alone or in combination. Moreover, among the patients who used T, many had liver cancer and there were some episodes wherein the effectiveness of the diuretics had not been noted in the patients' charts; thus, the data could not be collected. Therefore, multicenter prospective studies on this subject should be conducted at the earliest, with an analysis of the efficacy and clinical tolerance of T alone in this indication, or even in a randomized therapeutic trial.

## Conclusion

In this study, the effectiveness of diuretics on pedal edema was observed by patients in 57.1%–90.9% of episodes and by physicians in 50.0%–71.8% of episodes. Regarding the secondary outcome—differences in assessments between physicians and patients—the statistical analysis indicated that the patients' and the physicians' evaluations largely matched. The treatment of pedal edema improves patient symptoms, resulting in improved QOL. Therefore, it is important that symptoms improve according to the patients' assessment.

We found no statistically significant differences in the effectiveness of the various diuretics for treating pedal edema. However, according to the evaluations by patients and physicians, the ratio of patients for which there was an improvement in pedal edema was relatively high, at 62.5%–90.9%, for both T monotherapy and T combined with other diuretics. Further studies are necessary to collect additional data to evaluate the effects of T on pedal edema and verify our results.

## Ethical Approval

This study was approved by the ethics review committee at Ashiya Municipal Hospital (approval no. 91).

## Funding Information

No funding was received for this article.

## Abbreviations Used

A: azosemide
CI: confidence interval
F: furosemide
L: loop diuretics
MD: physicians
ND: no data
OR: odds ratio
Pt: patients
S: spironolactone
SD: standard deviation
T: tolvaptan
V2: vasopressin type 2
